# Diaminoquinazoline MMV675968 from Pathogen Box inhibits *Acinetobacter baumannii* growth through targeting of dihydrofolate reductase

**DOI:** 10.1038/s41598-019-52176-8

**Published:** 2019-10-30

**Authors:** Warangkhana Songsungthong, Suganya Yongkiettrakul, Louise E. Bohan, Eric S. Nicholson, Sunisa Prasopporn, Pimchai Chaiyen, Ubolsree Leartsakulpanich

**Affiliations:** 1 Biomolecular Analysis and Application Laboratory, National Center for Genetic Engineering and Biotechnology (BIOTEC), Pathum Thani, 12120 Thailand; 2 School of Biomolecular Science and Engineering, Vidyasirimedhi Institute of Science and Technology (VISTEC), Rayong, 21210 Thailand; 3 Present Address: UCD School of Biomolecular and Biomedical Science, University College Dublin, Dublin, 4 Ireland; 4 Present Address: Biology Department, Earlham College, Indiana, 47374 USA; 5 Present Address: Department of Pharmacology, Faculty of Medicine, Siriraj Hospital, Mahidol University, Bangkok, 10700 Thailand

**Keywords:** Drug discovery, Microbiology, Molecular biology

## Abstract

Antibiotic resistance in *Acinetobacter baumannii* is a major global health threat. New drugs with novel chemical structures are needed to overcome a myriad of resistance mechanisms in *A. baumannii*. In this study, we screened an open-source Pathogen Box library for anti-*A. baumannii* compounds. Compound MMV675968 (a diaminoquinazoline analog) was the only non-reference compound found to inhibit the growth of all four *A. baumannii* test strains with IC_50_ of 0.6–2.7 μM, IC_90_ of 0.7–3.9 μM, and MIC of 1.6–10 μM. We showed that MMV675968 targeted *A. baumannii* dihydrofolate reductase (*Ab*DHFR) as determined by an *E. coli* surrogate whose growth was dependent on *Ab*DHFR function and by an *in vitro* DHFR activity assay. Additionally, chemical scaffolds of DHFR inhibitors that are effective as antibiotics against *A. baumannii* were identified using an *in vitro* DHFR activity assay and *A. baumannii* growth inhibition. MMV675968 was the most potent among DHFR inhibitors tested in inhibiting *A. baumannii* growth. This study shows for the first time that MMV675968 inhibits *A. baumannii* growth via selective inhibition of *Ab*DHFR and is therefore a promising scaffold for further antibiotic development against *A. baumannii*.

## Introduction

*Acinetobacter baumannii*, a Gram-negative opportunistic pathogen, is a leading cause of nosocomial infection worldwide^1^. It has developed resistance to multiple classes of antibiotics with strains resistant to all commercially available antibiotics^1–4^. *A. baumannii* possesses an array of resistance mechanisms such as downregulation of porin, mutations in drug targets and altered expression of β-lactamases, aminoglycoside-modifying enzymes, and drug efflux pumps^5,6^. Treatment failure is common in *A. baumannii* infection owing to a lack of effective alternate antibiotics against this multidrug-resistant pathogen, responsible for up to 50% mortality among infected patients^1^. Discovery of new drug candidates and understanding of their mechanisms of action is direly needed to tackle the problem of resistance in this pathogenic organism.

The Open-source Pathogen Box consists of 400 compounds known to be active against a number of pathogens responsible for neglected tropical diseases and have low toxicity (www.pathogenbox.org). Pathogen Box contains compounds with diverse chemical scaffolds distinct from currently available antibiotics, making the Pathogen Box library a promising source of compounds for novel antibiotics discovery. The mechanisms of action of these compounds are mostly unknown but crucial for hit-to-lead development in a drug discovery process, thereby worth investigating further^7,8^. In this study, we used Pathogen Box compounds to screen for those with anti-*A. baumannii* activity and investigated the mode of action of the most promising bioactive candidate.

## Results

### MMV675968 inhibits the growth of *A. baumannii* strains

**Figure 1:**
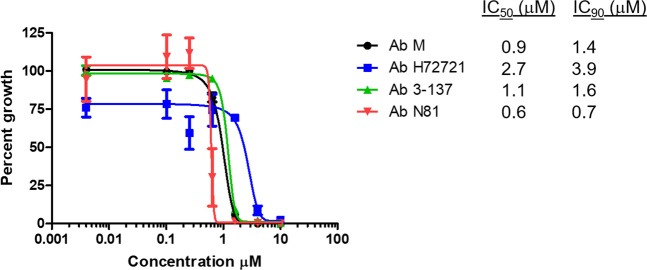
Growth inhibitory curve of MMV675968. Four strains of *A. baumannii*, namely the Mahidol strain (Ab M, circle), the H72721 strain (Ab H72721, square), the 3-137 strain (Ab 3-137, triangle), and the Naval-81 strain (Ab N81, inverted triangle) were incubated with various concentrations of MMV675968 for 18 hours. Growth was monitored using OD_600_. Percent growth was calculated relative to untreated *A. baumannii*, which was set to 100%. The plot shows mean ± standard error of the mean (SEM) from three independent experiments.

Initially, we screened the Pathogen Box library (www.pathogenbox.org) for compounds that inhibit the growth of *A. baumannii* Mahidol and Naval-81 strains, an environmental and clinical strain, respectively. Using the Clinical and Laboratory Standards Institute (CLSI) microdilution method, the majority of the compounds exhibited no significant growth inhibitory activity at 10 μM (Table S1). Five compounds inhibited *A. baumannii* growth to less than 50% of untreated (Table 1). However, of the five compounds with inhibitory activities, auranofin, doxycycline, levofloxacin-(-)-ofloxacin, and rifampicin are reference compounds with known antibacterial activity, with doxycycline and rifampicin completely inhibited the growth of both strains tested (Table 1). Compound MMV675968, a diaminoquinazoline, completely inhibited the growth of *A. baumannii* Naval-81 strain and inhibited *A. baumannii* Mahidol strain to 25% in the primary screen (Table 1). We then used various concentrations of MMV675968 to determine growth inhibition curve against four strains of *A. baumannii*, namely, Mahidol, Naval-81, H72721, and 3–137 strains. IC_50_, IC_90_, and MIC values were 0.6–2.7 μM (0.22–0.97 mg/L), 0.7–3.9 μM (0.25–1.40 mg/L), and 1.6–10 μM (0.58–3.60 mg/L), respectively (Fig. 1), which are comparable to corresponding values of effective antibiotics against *A. baumannii*^9–11^.

**Table 1:** Compounds (10 µM) in Pathogen Box that inhibited the growth of two *A. baumannii* (*Ab*) strains – the Mahidol strain and the Naval-81 strain to less than 50% of that of untreated (100%).

No.	Trivial Name	Compound ID	Percent Growth ± SD of	
			*Ab* Naval-81	*A*b Mahidol
1	Levofloxacin (-)-ofloxacin	MMV687798	38 ± 46	0 ± 0
2	Rifampicin	MMV688775	0 ± 0	0 ± 1
3	Doxycycline	MMV000011	0 ± 0	0 ± 0
4	—	MMV675968	0 ± 0	25 ± 51
5	Auranofin	MMV688978	28 ± 26	33 ± 31

Mean ± standard deviation (SD) from at least three independent experiments are shown.

### MMV675968 selectively inhibits *A. baumannii* DHFR

**Figure 2:**
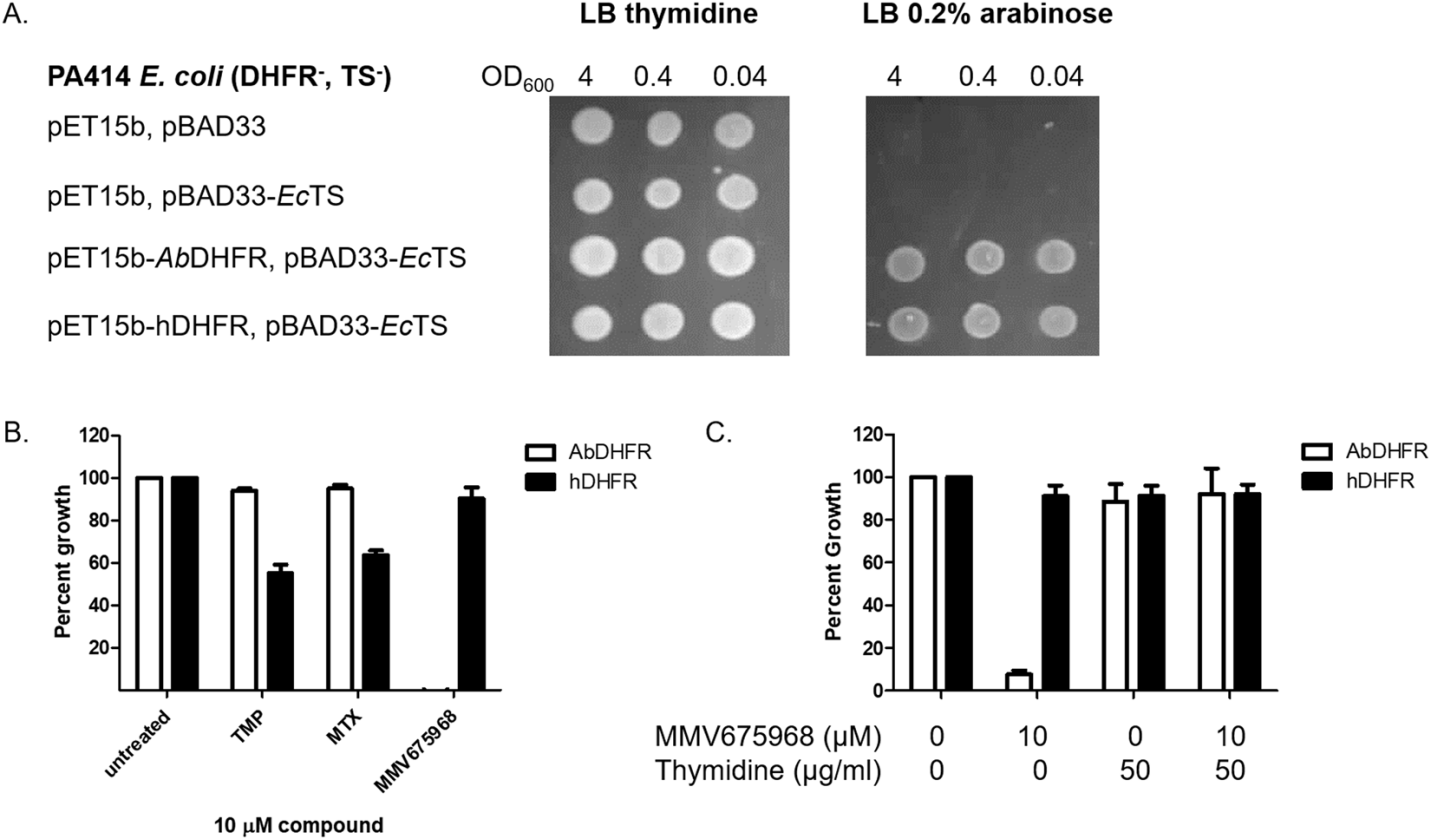
MMV675968 inhibits *Ab*DHFR but not hDHFR in an *E. coli* model. (**A**) *Ab*DHFR and hDHFR can complement for the loss of *Ec*DHFR. Cultures of *E. coli* PA414 (DHFR^−^, TS^−^) carrying empty plasmids or plasmids expressing *Ec*TS and either *Ab*DHFR or hDHFR were spotted onto LB plate supplemented with 50 µg/ml thymidine or 0.2% arabinose. A representative picture from three independent experiments is shown. (**B**) MMV675968 inhibits the growth of *E. coli* model expressing *Ab*DHFR not hDHFR when grown without thymidine supplement. (**C**) Thymidine supplement rescues growth inhibition of *E. coli* surrogate expressing *Ec*TS and *Ab*DHFR by MMV675968. *E. coli* PA414 expressing *Ec*TS and either *Ab*DHFR or hDHFR were incubated with 10 µM of DHFR inhibitors for 18 hours. Growth was monitored using OD_600_. Percent growth was calculated relative to untreated which was set at 100%. The graph shows mean ± SEM from three independent experiments.

MMV675968 inhibits DHFR of *Cryptosporidium*, *Plasmodium*, and *Escherichia coli*^12–14^. We hypothesized that MMV675968 might have a similar mode of action in *A. baumannii*. In order to test this notion, we used *E. coli* PA414 strain, which lacks dihydrofolate reductase (DHFR) and thymidylate synthase (TS) of thymidylate cycle required for thymidine synthesis hence making the strain a thymidine auxotroph^15^, as a model to test DHFR target specificity. The *E. coli* surrogate was co-transformed with plasmids expressing *E. coli* TS (*Ec*TS) and either *Ab*DHFR or human DHFR (hDHFR). When *Ec*TS was expressed together with *Ab*DHFR or hDHFR, *E. coli* PA414 was able to grow in the absence of thymidine (Fig. 2A), indicating that *Ab*DHFR and hDHFR could function in *E. coli* and can complement for the loss of *Ec*DHFR. The growth of the *E. coli* surrogates were then tested in the presence of MMV675968, trimethoprim (TMP), a bacterial DHFR inhibitor, or methotrexate (MTX), a known hDHFR inhibitor^16,17^. MMV675968 (10 µM) was able to inhibit the growth of the *E. coli* surrogate expressing *Ab*DHFR but not that of surrogate expressing hDHFR (Fig. 2B); conversely, TMP and MTX inhibited the growth of the *E. coli* surrogate expressing hDHFR but not *Ab*DHFR (Fig. 2B). These data suggest that 1) MMV675968 is more effective in inhibiting *Ab*DHFR than TMP and MTX, 2) MMV675968 is less effective in inhibiting hDHFR than MTX and TMP, and 3) MMV675868 is likely to be more specific to *Ab*DHFR than hDHFR since expression levels of *Ab*DHFR and hDHFR in *E. coli* PA414 were comparable (Fig. S2). In addition, thymidine supplement was able to rescue growth inhibition by MMV675968 (Fig. 2C), confirming that thymidylate cycle is the target of MMV675968 in *E. coli* surrogate.

**Figure 3:**
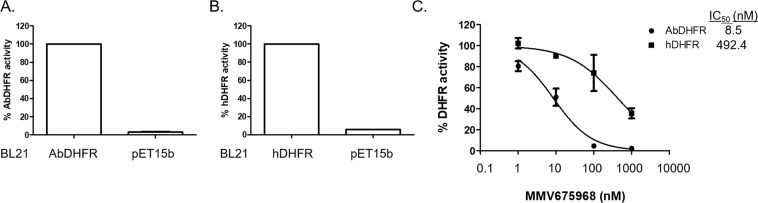
MMV675968 selectively inhibits *Ab*DHFR, not hDHFR, in an *in vitro* DHFR activity assay. DHFR activity present in lysate of BL21(DE3) containing pET15b and that overexpressing *Ab*DHFR (**A**) or hDHFR (**B**) were determined by measuring reduction of A_340_ and compared. (**C**) MMV675968 prefers *Ab*DHFR to hDHFR as target. *Ab*DHFR or hDHFR activity with various concentrations of MMV675968 was determined. DHFR activity of DMSO-treated lysate of BL21(DE3) overexpressing *Ab*DHFR or hDHFR was set to 100%. DHFR activity in the presence of inhibitors was calculated relative to DMSO-treated. The graphs show mean ± SEM from at least three independent experiments.

In addition to testing MMV675968 inhibition of *Ab*DHFR in an *E. coli* model, we also tested whether MMV675968 directly inhibited *Ab*DHFR and hDHFR in an enzyme activity assay. DHFR activities of lysate of *E. coli* BL21(DE3) harboring pET15b and that overexpressing *Ab*DHFR or hDHFR were compared. Using equal amount of total protein, DHFR activity present in the lysate of *E. coli* BL21(DE3) containing pET15b accounted for only 3% and 6% of that detected in the lysate of *E. coli* BL21(DE3) overexpressing *Ab*DHFR and hDHFR, respectively (Fig. 3A,B). These results suggest that most of DHFR activity measured in *E. coli* BL21(DE3) expressing *Ab*DHFR and hDHFR comes from overexpressed enzymes not endogenous *Ec*DHFR. We found that MMV675968 inhibited *Ab*DHFR and hDHFR in a dose dependent manner with IC_50_ values of 8.5 nM and 492.4 nM, respectively (Fig. 3C), confirming that MMV675968 prefers *Ab*DHFR to hDHFR as target.

### Structure-function relationship reveals MMV675968 as a potent antifolate scaffold targeting *A. baumannii*

**Figure 4:**
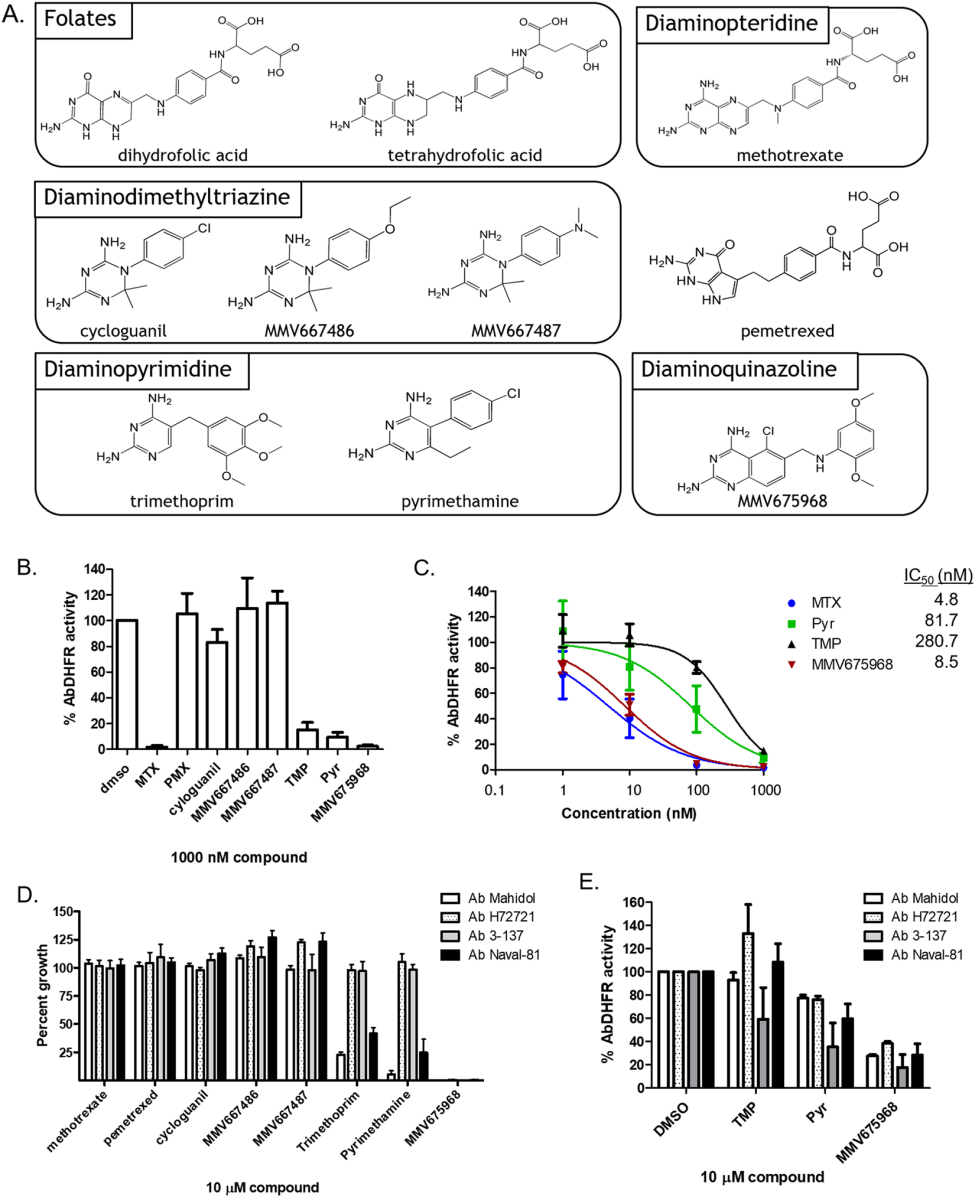
Structures of DHFR inhibitors and their activity in inhibiting *Ab*DHFR *in vitro*, *in vivo*, and in inhibiting growth of *A. baumannii*. (**A**) Structures of folates and DHFR inhibitors used in this study. DHFR inhibitors are divided into four major classes: (1) diaminopteridine - methotrexate, (2) diaminodimethyltriazine - cycloguanil, MMV667486, and MMV667487, (3) diaminopyrimidine - trimethoprim and pyrimethamine, and (4) diaminoquinazoline - MMV675968. (**B**) *In vitro Ab*DHFR activity in the presence of 1000 nM compounds. (**C**) Dose response curve of selected compounds and their anti-*Ab*DHFR activity. (**D**) *A. baumannii* growth inhibitory activity of 10 μM of DHFR inhibitors. Four strains of *A. baumannii*, namely the Mahidol strain (Ab Mahidol), the H72721 strain (Ab H72721), the 3-137 strain (Ab 3–137), and the Naval-81 strain (Ab Naval-81) were incubated with 10 µM of various inhibitors. (**E**) *Ab*DHFR activity of four *A. baumannii* strains after treatment with 10 µM for 90 minutes. Percent *Ab*DHFR activity or percent growth was calculated compared with untreated, which was set to 100%. The graphs show mean ± SEM from at least three independent experiments.

The chemical structures of DHFR inhibitors vary with different chemical scaffolds possessing different efficacy toward different targets. To determine which chemical scaffolds are the most effective antibiotic candidates against *A. baumannii*, MMV675968 and seven other antimicrobial DHFR inhibitors (Fig. 4A) were tested for their efficacy in inhibiting *Ab*DHFR *in vitro* and *in vivo* and in inhibiting *A. baumannii* growth. *Ab*DHFR activity in the presence of 1000 nM inhibitors were determined (Fig. 4B). At 1000 nM, PMX, cycloguanil, MMV667486, and MMV667487 showed little inhibitory activity, while MTX, TMP, PYR, and MMV675968 strongly inhibited *Ab*DHFR activity *in vitro* (Fig. 4B). MTX, TMP, PYR, and MMV675968 were chosen for further IC_50_ determination. MTX and MMV675968 potently inhibited *Ab*DHFR *in vitro* with IC_50_ values of 4.8 nM and 8.5 nM, respectively (Fig. 4C). PYR and TMP, both of which are diaminopyrimidine analogs, were less effective in inhibiting *Ab*DHFR *in vitro* with IC_50_ values of 81.7 and 280.7 nM, respectively (Fig. 4C).

Eight DHFR inhibitors were then tested for their *A. baumannii* growth inhibitory activity. MMV675968 was the only compound that completely inhibited the growth of all four *A. baumannii* strains (Fig. 4D). Even though MTX strongly inhibited *Ab*DHFR *in vitro* (Fig. 4C), it did not inhibit the growth of any *A. baumannii* strains tested (Fig. 4D), implying that MTX may not be able to reach *Ab*DHFR target within *A. baumannii* cells. TMP and pyrimethamine (PYR) inhibited the growth of two strains, namely, Mahidol and Naval-81 strains, but not the H72721 and 3–137 strains (Fig. 4D). All four strains share identical *Ab*DHFR polypeptide sequence deduced from DNA sequences of amplified gene fragments (Fig. S3), indicating that differences in PYR and TMP sensitivities among the test *A. baumannii* strains are due to factor(s) not related to *Ab*DHFR inhibition.

To further confirm that MMV675968 targets *Ab*DHFR in *A. baumannii*, we determined whether *Ab*DHFR activity of each *A. baumannii* strain decreased upon treatment with DHFR inhibitors. *A. baumannii* strains were treated with 10 µM TMP, PYR, or MMV675968 for 90 minutes. Normalized lysate was then tested for DHFR activity. All four *A. baumannii* strains showed reduced DHFR activity upon MMV675968 treatment compared with untreated cells (Fig. 4E), confirming that MMV675968 targets *Ab*DHFR *in vivo*. Higher *Ab*DHFR inhibitory activity of MMV675968 in this assay compared with other inhibitors tested is probably due to a combination of differences in cell permeability, drug efflux rate, and *Ab*DHFR inhibitory efficacy.

## Discussion

In this study, an open-source Pathogen Box was screened for bioactive compounds against *A. baumannii*. The advantages of using such library are the availability of pure compounds, the knowledge of chemical structures, and the access to related information such as toxicity and pharmacokinetics properties (www.pathogenbox.org). The open source format is expected to jump-start the discovery of new antibiotics urgently needed to combat drug resistant organisms. From the Pathogen Box, compound MMV675968 was discovered to be an effective inhibitor of *A. baumannii* growth with IC_50_, IC_90_, and MIC values comparable to known effective antibiotics against *A. baumannii* (Table 1, Fig. 1)^9–11^.

MMV675968 inhibits DHFR, an essential enzyme in the folate biosynthetic pathway, in *Cryptosporidium*, *Plasmodium*, and *E. coli*^12–14^. This study is the first to show that MMV675968 has anti-*A. baumannii* activity through inhibition of *Ab*DHFR as determined by an *E. coli* surrogate model and an *in vitro* DHFR activity assay. MMV675968 inhibits *Ab*DHFR more effectively than hDHFR, with 58 fold selectivity (Fig. 3C), consistent with biochemical data from others that this compound is 83 fold more effective in inhibiting *E. coli* DHFR than hDHFR^12^. Since structures of bacterial DHFRs and mammalian DHFRs are clustered in different phylogenetic groups^18^, the presence of selective bacterial DHFR inhibitors like trimethoprim is possible.

There are at least four major classes of DHFR inhibitors, namely, diaminopteridine, diaminodimethyltriazine, diaminopyrimidine, and diaminoquinazoline (Fig. 4A)^19^. MTX, an anti-cancer diaminopteridine, showed strong *in vitro Ab*DHFR inhibition (Fig. 4B,C) but did not show *Ab*DHFR inhibitory activity in *E. coli* surrogate (Fig. 2B) and did not have antibacterial activity against *A. baumannii* (Fig. 4D), suggesting that MTX may not reach *Ab*DHFR target in both *E. coli* surrogate and in *A. baumannii*. It is possible that MTX cannot permeate *E. coli* and *A. baumannii* cells, is degraded by enzymes, and/or is pumped out via efflux pump(s) such as AcrAB/TolC efflux pump, which is shown to export MTX, rendering MTX ineffective against *E. coli*^20^.

Diaminodimethyltriazines such as cycloguanil, MMV667486, and MMV667487, which are *Plasmodium* DHFR inhibitors^19,21,22^, did not inhibit *Ab*DHFR *in vitro* or show any growth inhibitory activity against *A. baumannii* (Fig. 4B,D), highlighting structural differences between *Ab*DHFR and *Plasmodium* DHFR.

Diaminopyrimidines such as PYR and TMP are inhibitors of *Plasmodium* and bacterial DHFR, respectively^19^. While being moderately effective in inhibiting *Ab*DHFR *in vitro* (Fig. 4B,C), TMP did not inhibit *Ab*DHFR in *E. coli* surrogate (Fig. 2B), possibly due to compound permeability and/or compound efficacy against *Ab*DHFR. Diaminopyrimidines inhibited the growth of only two of the four test *A. baumannii* strains (Fig. 4D). The reason for differential diaminopyrimidine sensitivity among *A. baumannii* strains was not due to *Ab*DHFR sequence (Fig. S3) and requires further investigation. Various *A. baumannii* strains may differ in terms of levels of *Ab*DHFR expression, alteration of cell permeability, and/or differences in efflux pump.

MMV675968, a diaminoquinazoline, inhibited *Ab*DHFR 33 fold better than TMP (Fig. 4C), inhibited the growth of both TMP-sensitive and -resistant *A. baumannii* strains (Fig. 4D), and inhibited DHFR activity in *A. baumannii* (Fig. 4E). MMV675968 is therefore more effective in inhibiting *Ab*DHFR than TMP and can bypass TMP- and PYR-resistance mechanism present in *A. baumannii* H72721 and the 3–137 strains. Since *Acinetobacter* species cannot salvage thymine or thymidine^23–25^, enzymes of thymidylate cycle become even more critical to its survival and could serve as promising drug targets. MMV675968 and its diaminoquinazoline analogs are, therefore, worth investigating further as antibiotic candidates against *A. baumannii*.

DHFR, an essential enzyme that converts dihydrofolate to tetrahydrofolate, is a validated drug target in bacteria and protozoan parasites. There may be concerns that *Ab*DHFR might not be an effective drug target because resistant mutations can arise and lead to drug resistance. However, since DHFR active site needs to accommodate dihydrofolate substrate for its essential function, there should be a limit number of mutations that *Ab*DHFR active site can tolerate. Designing a DHFR inhibitor to stay in the substrate space has proved to be beneficial in making the P218 compound bind to and inhibit both wild type and pyrimethamine-resistant mutant of *Plasmodium* DHFR and effectively inhibits both wild type and mutant parasites^26^. It is therefore theoretically possible to use *Ab*DHFR as a drug target despite possible rise of resistant mutations.

Another strategy to prevent rapid emergence of drug resistance is the use of drug combinations, such as using DHFR inhibitors in combination with dihydropteroate synthase (DHPS) inhibitors, e.g. trimethoprim-sulfamethoxazole combination (Bactrim) and pyrimethamine-sulfadoxine combination (Fansidar). Using DHPS inhibitors in combination with MMV675968 or its analogs should help reduce the required doses and forestall resistance.

The *E. coli* surrogate expressing exogenous *Ab*DHFR or hDHFR constructed in this study can be used for screening other compound libraries for selective inhibitors of *Ab*DHFR in an antibiotic discovery research program. Model organisms lacking endogenous DHFR activity have been used to study the activity of exogenously expressed DHFR from pathogenic species and for inhibitor screening^27–29^. The advantages of such surrogate models are the ease and safety of handling model organisms rather than the pathogenic organisms of interest. However, one caveat of using model organisms for studying target enzymes is the vast differences in cell permeability between species. For example, compounds that are active against a *Plasmodium* enzyme expressed in yeast may not be able to penetrate *Plasmodium* membrane. However, since *E. coli* and *A. baumannii* are both Gram-negative bacteria, they share similar cell wall/cell membrane structure, making the results from the surrogate assay developed in this study easier to translate from one organism to the other.

In summary, we identified a diaminoquinazoline MMV675968 that could serve as a starting compound for drug development against multidrug-resistant *A. baumannii*. Further comparative studies to understand enzyme kinetics and structures of *Ab*DHFR and hDHFR and studies on *in vivo* efficacy, toxicity, pharmacokinetics, and pharmacodynamics can provide insights into improving the starting compound into more effective and/or more selective antibiotic.

## Methods

### Bacteria strains and culture conditions

*A. baumannii* Mahidol strain was isolated from soil as described previously^30^, and multidrug-resistant clinical Naval-81, 3–137 (OIFC137), and H72721 isolates were from BEI Resources, NIAID, NIH, USA^31,32^. *E. coli* PA414 strain carrying null mutations in *folA* and *thyA* encoding dihydrofolate reductase (DHFR) and thymidylate synthase (TS), respectively was used as a host for expressing DHFR from other organisms and TS from *E. coli*^15^. *E. coli* and *A. baumannii* were grown in Luria Bertani (LB) broth supplemented as necessary with 50 μg/ml thymidine, 100 μg/ml ampicillin, 10 μg/ml chloramphenicol, 0.2% arabinose, or their combination and incubated with shaking at 37 °C.

### Growth inhibition assay

We performed bacterial growth inhibition assay according to the Clinical and Laboratory Standards Institute (CLSI) microdilution method^33^. In brief, compounds were solubilized and diluted in dimethyl sulfoxide (DMSO). Approximately 5 × 10^4^ colony forming units (CFUs) were incubated with 10 μM (unless otherwise indicated) of each compound in cation adjusted Mueller Hinton broth (CAMHB) (Beckton Dickinson, Franklin Lakes, NJ, USA) at 37 °C for 18 hours. Optical density at 600 nm (OD_600_) was measured using SpectraMax M5 (Molecular Devices, San Jose, CA, USA). OD_600_ of wells containing *A. baumannii* (either compound-treated or untreated) was subtracted with OD_600_ of blank. Percent growth of compound-treated *A. baumannii* of the same strain was calculated using the following formula:

$$(OD\,of\,compound\,treated\,strain{\rm{\#}}1\times 100)/(OD\,of\,DMSO\,treated\,strain{\rm{\#}}1)$$

Percent growth of each DMSO-treated *A. baumannii* strain was therefore 100%. Hits are defined as compounds that inhibit bacteria growth >50%. For IC_50_ and IC_90_ (50% and 90% inhibitory concentration) determination, *A. baumannii* strains were treated with various concentrations of test compound and cultured according to the microdilution method above. Minimum inhibitory concentration (MIC) is defined as the lowest concentration that completely inhibits bacteria growth as determined by unaided eye^33^.

### Construction of expression plasmids

*Abdhfr* was amplified from clarified cell lysates of four strains of *A. baumannii* using Phusion DNA polymerase (New England Biolabs, Ipswich, MA, USA) and primers 5′ctggtgccgcgcggcagccatATGGCATGGCAAAATGTAG3′ and 5′tcgggctttgttagcagccggatccTTATTTTTTATAAGTGGCAAATTCG3′ (lower case denoting sequence homologous to pET15b plasmid), and human DHFR from pL0035^34^ using primers 5′ctggtgccgcgcggcagccatATGGTTGGTTCGCTAAAC3′ and 5′tcgggctttgttagcagccggatccTTAATCATTCTTCTCATATACTTCAAATTTG3′. Amplicons were inserted into NdeI and BamHI digested pET15b plasmid using a Gibson Assembly kit (New England Biolabs). The thymidylate synthase gene (*thyA, ts*) was amplified from lysate of *E. coli* DH5α strain using Phusion DNA polymerase (New England Biolabs, Ipswich, MA, USA) and primers 5′ctagcggagctcggagtgaaacgATGAAACAGTATTTAGAACTGATGC3′ and 5′ gcctgcaggtcgactctagaTTAGATAGCCACCGGCGC3′ (lower case denoting sequence homologous to pBAD33 plasmid). Amplicon was inserted into SacI and XbaI sites of pBAD33 using the Gibson Assembly kit (New England Biolabs, Ipswich, MA, USA). Cloned plasmids were sequenced by 1^st^ Base DNA sequencing service (Singapore) and nucleotide sequences were deposited at GenBank, accession numbers: MH152688, MH152689, MH152690, and MH152691.

### Agar plate growth assay

*E. coli* PA414 strain carrying various combination of expression plasmids was grown overnight at 37 °C with shaking in LB broth supplemented with 50 μg/ml thymidine, 10 μg/ml chloramphenicol, and 100 μg/ml ampicillin. Cells from overnight cultures were pelleted by centrifugation, washed with fresh medium and the OD_600_ adjusted to 4.0. Ten microliters of undiluted, 10-fold and 100-fold serially diluted cells were spotted onto LB agar plates supplemented with necessary antibiotics and 50 μg/ml thymidine or 0.2% arabinose and incubated at 37 °C overnight.

### Overexpression of *Ab*DHFR and hDHFR in *E. coli* BL21(DE3)

Overnight cultures of *E. coli* BL21(DE3) carrying pET15b, pET15b-*Ab*DHFR, and pET15b-hDHFR were diluted into fresh LB supplemented with 100 µg/ml ampicillin and grown with shaking at 37 °C for 3 hours. After addition IPTG to final concentration of 40 mM, the cultures were grown overnight with shaking at 16 °C. Bacteria cells were harvested by centrifugation and lysed by sonication. Protein concentration of clarified bacteria lysate was determined using Bradford assay (Biorad, Hercules, CA, USA) according to manufacturer’s protocol.

### DHFR activity assay

The assay was performed as previously described^26^. Briefly, 0.2 µg of total protein from lysate of *E. coli* containing pET15b or overexpressing *Ab*DHFR or 5 µg of total protein from lysate of *E. coli* containing pET15b or overexpressing hDHFR was added to reaction mixture containing 50 mM TES, pH 7.0, 75 mM β-mercaptoethanol, 1 mM EDTA, 1 mg/ml BSA, 0.1 mM NADPH, 0.1 mM dihydrofolate, and various concentrations of inhibitors to initiate the enzymatic reaction. Absorbance at 340 nm (A_340_) was monitored over 100 seconds. DHFR activity was measured as changes in A_340_ per minute.

### *Ex vivo Ab*DHFR activity assay

To determine whether *Ab*DHFR activity in *A. baumannii* cells is affected by DHFR inhibitors, overnight cultures of *A. baumannii* strains were harvested, OD_600_-adjusted to 5.6 OD/ml and treated with 10 µM inhibitors for 90 minutes. To determine DHFR activity in treated *A. baumannii*, cells were harvested, washed once with and resuspended in DHFR buffer (20 mM potassium phosphate buffer, pH 7.0, 0.1 mM EDTA, 10 mM DTT, 50 mM KCl, 20% glycerol). *A. baumannii* suspension was lysed by sonication, normalized for protein content, and used to initiate DHFR activity assay. DHFR activity in untreated *A. baumannii* was set to 100% activity. DHFR activity of inhibitor-treated *A. baumannii* was calculated relative to untreated control.

## Supplementary information

Supplementary Information

Supplementary Information

## References

[1] Munoz-Price LS, Weinstein RA (2008). *Acinetobacter* Infection. N. Engl. J. Med..

[2] Lu CL (2011). Antimicrobial susceptibilities of commonly encountered bacterial isolates to fosfomycin determined by agar dilution and disk diffusion methods. Antimicrob. Agents Chemother..

[3] Leite GC (2016). Antimicrobial Combinations against Pan-Resistant *Acinetobacter baumannii* Isolates with Different Resistance Mechanisms. PLoS One.

[4] Gales AC, Jones RN, Sader HS (2006). Global assessment of the antimicrobial activity of polymyxin B against 54 731 clinical isolates of Gram-negative bacilli: Report from the SENTRY antimicrobial surveillance programme (2001-2004). Clin. Microbiol. Infect..

[5] Bonomo RA SD (2006). Mechanism of multidrug resistance in *Acinetobacter* species and *Pseudomonas aeruginosa*. Clin. Infect. Dis..

[6] Peleg AY, Adams J, Paterson DL (2007). Tigecycline efflux as a mechanism for nonsusceptibility in *Acinetobacter baumannii*. Antimicrob. Agents Chemother..

[7] Bleicher KH, Böhm HJ, Müller K, Alanine AI (2003). Hit and lead generation: Beyond high-throughput screening. Nat. Rev. Drug Discov..

[8] Congreve M, Murray CW, Blundell TL (2005). Keynote review: Structural biology and drug discovery. Drug Discov. Today.

[9] Cheol Park G (2016). *In Vitro* Interactions of Antibiotic Combinations of Colistin, Tigecycline, and Doripenem Against Extensively Drug-Resistant and Multidrug-Resistant *Acinetobacter baumannii*. Ann Lab Med.

[10] Siricilla S (2017). A New Combination of a Pleuromutilin Derivative and Doxycycline for Treatment of Multidrug-Resistant *Acinetobacter baumannii* HHS Public Access. J Med Chem. April.

[11] The Clinical and Laboratory Standards Institute. *Performance Standards for Antimicrobial Susceptibility Testing CLSI supplement M100S*. *Clinical and Laboratory Standards Institute, Wayne, PA*. (2016).

[12] Nelson RG, Rosowsky A (2001). Dicyclic and tricyclic diaminopyrimidine derivatives as potent inhibitors of *Cryptosporidium parvum* dihydrofolate reductase: Structure-activity and structure-selectivity correlations. Antimicrob. Agents Chemother..

[13] Lau, H., Ferlan, J. T., Hertle Brophy, V., Rosowsky, A. & Sibley, C. H. Efficacies of Lipophilic Inhibitors of Dihydrofolate Reductase against Parasitic Protozoa. **45**, 187–195 (2001). 10.1128/AAC.45.1.187-195.2001 PMC90259 11120964

[14] Popov VM (2006). Analysis of complexes of inhibitors with *Cryptosporidium hominis* DHFR leads to a new trimethoprim derivative. Bioorganic Med. Chem. Lett..

[15] Ahrweiler PM, Frieden C (1988). Construction of a fol mutant strain of *Escherichia coli* for use in dihydrofolate reductase mutagenesis experiments. J. Bacteriol..

[16] Goodsell DS (1999). The molecular perspective: methotrexate. Oncologist.

[17] Rajagopalan PTR (2002). Interaction of dihydrofolate reductase with methotrexate: ensemble and single-molecule kinetics. Proc. Natl. Acad. Sci. USA.

[18] Hecht D, Tran J, Fogel GB (2011). Structural-based analysis of dihydrofolate reductase evolution. Mol. Phylogenet. Evol..

[19] Lele AC, Mishra DA, Kamil TK, Bhakta S, Degani MS (2016). Repositioning of DHFR Inhibitors. Curr. Top. Med. Chem..

[20] Kopytek SJ, Dyer JC, Knapp GS, Hu JC (2000). Resistance to methotrexate due to AcrAB-dependent export from *Escherichia coli*. Antimicrob. Agents Chemother..

[21] Foote SJ, Galatis D, Cowman AF (1990). Amino acids in the dihydrofolate reductase-thymidylate synthase gene of *Plasmodium falciparum* involved in cycloguanil resistance differ from those involved in pyrimethamine resistance. Proc. Natl. Acad. Sci. USA.

[22] Aroonsri A (2016). Identifying antimalarial compounds targeting dihydrofolate reductase-thymidylate synthase (DHFR-TS) by chemogenomic profiling. Int. J. Parasitol..

[23] Ovrebo S, Kleppe K (1973). Pyrimidine metabolism in *Acinetobacter calcoaceticus*. J. Bacteriol..

[24] Rajamani S (2018). Bioengineering of bacterial pathogens for noninvasive imaging and *in vivo* evaluation of therapeutics OPEN. Sci. Reports.

[25] Towner, K. The Genus *Acinetobacter*. In *The Prokaryotes* 746–758, 10.1007/0-387-30746-X_25 (Springer New York, 2006).

[26] Yuthavong, Y. *et al*. Malarial dihydrofolate reductase as a paradigm for drug development against a resistance-compromised target. 10.1073/pnas.1204556109 PMC3479511 23035243

[27] Talawanich Y, Kamchonwongpaisan S, Sirawaraporn W, Yuthavong Y (2015). Use of bacterial surrogates as a tool to explore antimalarial drug interaction: Synergism between inhibitors of malarial dihydrofolate reductase and dihydropteroate synthase. Acta Trop..

[28] Tirakarn S (2012). Cloning and heterologous expression of *Plasmodium ovale* dihydrofolate reductase-thymidylate synthase gene. Parasitol. Int..

[29] Wooden JM, Hartwell LH, Vasquez B, Sibley CH (1997). Analysis in yeast of antimalaria drugs that target the dihydrofolate reductase of *Plasmodium falciparum*. Mol. Biochem. Parasitol..

[30] Thotsaporn K, Sucharitakul J, Wongratana J, Suadee C, Chaiyen P (2004). Cloning and expression of p-hydroxyphenylacetate 3-hydroxylase from *Acinetobacter baumannii*: Evidence of the divergence of enzymes in the class of two-protein component aromatic hydroxylases. Biochim. Biophys. Acta - Gene Struct. Expr..

[31] Tien HC (2007). Multi-drug resistant *Acinetobacter* infections in critically injured Canadian forces soldiers. BMC Infect. Dis..

[32] Chan, A. P. *et al*. A novel method of consensus pan-chromosome assembly and large-scale comparative analysis reveal the highly flexible pan-genome of *Acinetobacter baumannii*. *Genome Biol*. **16** (2015). 10.1186/s13059-015-0701-6 PMC4507327 26195261

[33] The Clinical and Laboratory Standards Institute. *Methods for Dilution Antimicrobial Susceptibility Tests for Bacteria That Grow Aerobically*. (2018).

[34] Braks JAM, Franke-Fayard B, Kroeze H, Janse CJ, Waters AP (2006). Development and application of a positive-negative selectable marker system for use in reverse genetics in *Plasmodium*. Nucleic Acids Res..

