# Supplementary material for: Diaminoquinazoline MMV675968 from Pathogen Box inhibits Acinetobacter baumannii growth through targeting of dihydrofolate reductase
Source: Sci Rep. 2019 Oct 30;9:15625. doi: 10.1038/s41598-019-52176-8 (PMC6821926; doi:10.1038/s41598-019-52176-8)
Supplement: Supplementary file 1 — Supplementary Information [file 41598_2019_52176_MOESM1_ESM.pdf]

Diaminoquinazoline MMV675968 from Pathogen Box inhibits *Acinetobacter baumannii* growth through targeting of dihydrofolate reductase

Warangkhana Songsungthong, Suganya Yongkiettrakul, Louise E. Bohan, Eric S. Nicholson, Sunisa Prasopporn, Pimchai Chaiyen, Ubolsree Leartsakulpanich

Supplementary Information

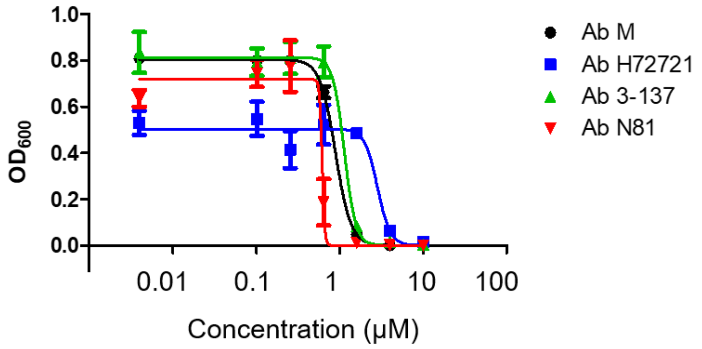

Fig. S1. Growth inhibitory curve of four *A. baumannii* strains by MMV675968. Growth (OD_{600}) of four *A. baumannii* strains upon treatment with various concentrations of MMV67568.

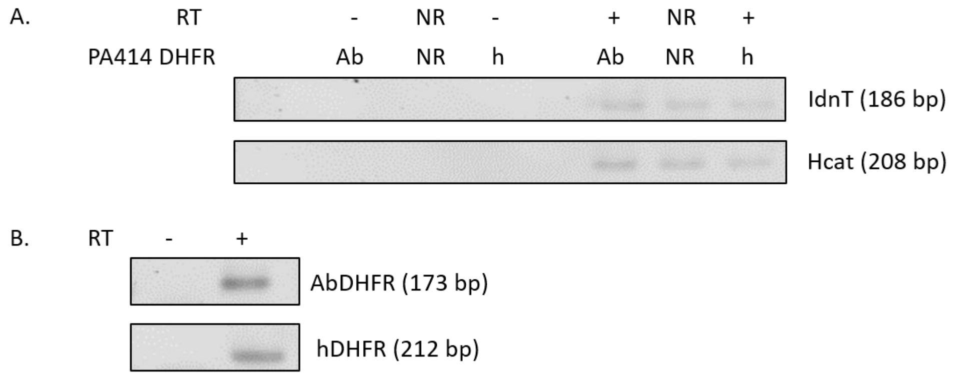

Fig. S2. Gene expression of *Ab*DHFR and hDHFR in *E. coli* PA414 surrogate. A) RT-PCR of reference genes – *idnT* and *hcaT* [1]. B) RT-PCR of *Ab*DHFR and hDHFR. Images were crop from different gels and arranged together. RT - reverse transcriptase. DHFR – dihydrofolate

reductase. *Ab* – *A. baumannii*. h – human. bp – base pairs. NR – this lane is not related to this study**.**

```
Red      - folate binding site
Blue     - NADPH binding site

AbN81DHFR        MAWQNVEVVHVVAMDKNHCIGKGNALPWHISADLKHFKEITQGGVVIMGRKTLESMGRAL     60
AbMDHFR          MAWQNVEVVHVVAMDKNHCIGKGNALPWHISADLKHFKEITQGGVVIMGRKTLESMGRAL     60
AbH72721DHFR     MAWQNVEVVHVVAMDKNHCIGKGNALPWHISADLKHFKEITQGGVVIMGRKTLESMGRAL     60
Ab3-137DHFR      MAWQNVEVVHVVAMDKNHCIGKGNALPWHISADLKHFKEITQGGVVIMGRKTLESMGRAL     60
                 ************************************************************

AbN81DHFR        PNRVNWVITRDINWHFDGVKIAYSIEDALNAALEDAKNTEKQALFIIGGGEIFKQTLSIA     120
AbMDHFR          PNRVNWVITRDINWHFDGVKIAYSIEDALNAALEDAKNTEKQALFIIGGGEIFKQTLSIA     120
AbH72721DHFR     PNRVNWVITRDINWHFDGVKIAYSIEDALNAALEDAKNTEKQALFIIGGGEIFKQTLSIA     120
Ab3-137DHFR      PNRVNWVITRDINWHFDGVKIAYSIEDALNAALEDAKNTEKQALFIIGGGEIFKQTLSIA     120
                 ************************************************************

AbN81DHFR        DRLELTHVDLDVQGDAHYPTIPSEFHKTASEQQVDEKSGTSFEFATYKK                169
AbMDHFR          DRLELTHVDLDVQGDAHYPTIPSEFHKTASEQQVDEKSGTSFEFATYKK                169
AbH72721DHFR     DRLELTHVDLDVQGDAHYPTIPSEFHKTASEQQVDEKSGTSFEFATYKK                169
Ab3-137DHFR      DRLELTHVDLDVQGDAHYPTIPSEFHKTASEQQVDEKSGTSFEFATYKK                169
                 *************************************************
```

Fig. S3. Amino acid sequence alignment of DHFR from four *A. baumannii* strains. The *dhfr* gene sequences were amplified from the four *A. baumannii* strains and cloned into the pET15b plasmid. The resulting plasmids were subjected to Sanger sequencing. The DNA sequences were translated to protein sequences using ExPASy translate tool [2] and multiply-aligned using CLUSTAL Omega [3]. Residues predicted to bind to folate (red) or NADPH (blue) by NCBI BLAST and NCBI conserved domain [4] are shown.

Materials and methods

Determination of gene expression level in *E. coli* PA414 using RT-PCR.

Overnight cultures of *E. coli* PA414 containing a combination of pBAD33-EcTS and either pET15b-*Ab*DHFR or pET15b-hDHFR were diluted to OD 0.1 into LB supplemented with 100 µg/ml ampicillin, 10 µg/ml chloramphenicol, and 0.2% arabinose, grown at 37 °C with shaking overnight. RNA was extracted from *E. coli* PA414 using RNeasy Plus Mini kit according to the manufacturer's protocol (Qiagen, Dusseldorf, Germany), DnaseI (New England Biolabs, Ipswich, MA) treated, purified using phenol/chloroform/isoamyl alcohol precipitation, and precipitated using ethanol precipitation. Reverse transcription was performed to obtain cDNA using M-MuLV reverse transcriptase according to the manufacturer's protocol (New England Biolabs, Ipswich, MA) using random hexamers as primers. PCR was performed using cDNA as template and primers specific to the following genes: *hcaT* (5′ggattggtttaacgccagaa3′ and 5′ccaatcatcaccagcatcag3′), *idnT* (5′ tcccgctttaatggtactgg 3′ and 5′ acggcgttaatggctaacac 3′), *Abdhfr* (5′gcagaccgtcttgagctcac 3′ and pET15b-specific primer 5′ ctttgttagcagccggatcc), and *hdhfr* (5′gccatgaatcacccaggcc 3′ and pET15b-specific primer 5′ ctttgttagcagccggatcc). The *hcat* and *idnT* genes were shown to be invariant during different growth phases of *E. coli*, thereby can be used as reference genes for expression study [1].

References

1. Zhou, K. *et al.* Novel reference genes for quantifying transcriptional responses of Escherichia coli to protein overexpression by quantitative PCR. *BMC Mol. Biol.* **12**, (2011).
2. Gasteiger, E. *et al.* ExPASy: The proteomics server for in-depth protein knowledge and analysis. *Nucleic Acids Res.* **31**, 3784–8 (2003).
3. Sievers, F. *et al.* Fast, scalable generation of high-quality protein multiple sequence alignments using Clustal Omega. *Mol. Syst. Biol.* **7**, 539 (2011).
4. Marchler-Bauer, A. *et al.* CDD: NCBI's conserved domain database. *Nucleic Acids Res.* **43**, D222–D226 (2015).
